# The genes associated with early-onset Alzheimer’s disease

**DOI:** 10.18632/oncotarget.23738

**Published:** 2017-12-15

**Authors:** Meng-Hui Dai, Hui Zheng, Ling-Dan Zeng, Yan Zhang

**Affiliations:** ^1^ Department of Neurology, Union Hospital, Tongji Medical College, Huazhong University of Science and Technology, Wuhan, 430022, China; ^2^ Department of Radiology, Union Hospital, Tongji Medical College, Huazhong University of Science and Technology, Wuhan, 430022, China

**Keywords:** Alzheimer’s disease, early-onset AD, APP, PSEN1, PSEN2

## Abstract

Alzheimer's disease (AD) is a progressive neurodegenerative disorder that accounts for the most cases of dementia, which is characterized by the deposition of dense plaques of amyloid beta (Aβ) plaques and neurofibrillary tangles consisting of hyperphosphorylated tau. The two main types of AD can be classified as early-onset AD (EOAD, onset < 65 years) and late-onset AD (LOAD, onset ≥ 65 years). Evidence from family and twin studies indicate that genetic factors are estimated to play a role in at least 80% of AD cases. The first milestone with linkage analysis revealed the mutations in *APP*, *PSEN1*, and *PSEN2* genes that cause EOAD. But pathogenic mutations in these three genes can only explain a small fraction of EOAD families. The additional disease-causing genes have not yet been identified. This review provides an overview of the genetic basis of EOAD and the relationship between the functions of these risk genes and the neuropathologic features of AD. A better understanding of genetic mechanisms underlying EOAD pathogenesis and the potentially molecular mechanisms of neurodegeneration will lead to the development of effective diagnosis and treatment strategies for this devastating disease.

## INTRODUCTION

Alzheimer’s disease (AD) is a neurodegenerative disease and the most common form of dementia in elderly people, leading to progressive and widespread damage to the brain and, ultimately, death [1]. Worldwide, 47.5 million people are living with dementia, and nearly 7.7 million new diagnoses are made every year [2]. The total number of people with dementia is projected to 75.6 million by 2030 and almost tripling to 135.5 million by 2050 [2]. The alarming rise of AD will impose a mounting financial and social burden worldwide. We urgently need to development of effective treatment strategies to prevent, delay the onset, slow the progression, or improve the symptoms of AD [3].

The key pathological changes observed in AD brain tissue are amyloid beta (Aβ) peptide deposited and neuritic plaques, hyperphosphorylated tau protein and neurofibrillary tangles [4, 5]. Additional changes include microgliosis and loss of neurons, white matter and synapses. At present, the exact etiology of AD is unclear. AD is considered as a complex disease, resulted from the complicated interactions between the multiple factors, such as the age, education, genetic, and environmental factors. The familial aggregation of AD shows that genetic factors may play a vital essential role in the development of the disease [6–8]. Based on its age of onset, AD is classified into early-onset AD (EOAD, onset < 65 years) and late-onset AD (LOAD, onset ≥ 65 years). The proportion of EOAD in all AD cases is between 5% and 10%. LOAD is a highly polygenic disease. By contrast, EOAD or autosomal dominantly inherited AD is substantially or even entirely genetically determined frequently associated with genetic causes [7]. EOAD is generally associated with a more rapid rate of progression, so better understanding of genetic mechanisms underlying EOAD pathogenesis will lead to the development of effective diagnosis and treatment strategies.

Three causal genes, which encode proteins involved in amyloid precursor protein (APP) breakdown and Aβ generation, have been identified so far for EOAD, including the *APP* gene on chromosome 21 [9], the presenilin 1 (*PSEN1*) gene on chromosome 14 [10], and presenilin 2 (*PSEN2*) on chromosome 1 [11]. AD-linked mutations in these three genes exhibit high penetrance (>85%), are mostly autosomal dominantly inherited, and lead with certainty to early-onset disease. Consequently, they are considered ‘diagnostic biomarkers’ of the disease [5]. However, mutations in these three genes can only explain a small fraction (5 to 10%) of EOAD cases [7, 12]; over 50% of Mendelian cases and most of sporadic EOAD cases remain genetically unexplained [7, 13–15]. In this review, we provide a summary of the genetic basis of EOAD, the relationship between these risk genes and the pathogenesis of EOAD.

### The genetic architecture of EOAD

#### APP

#### The gene and protein structure of APP

The gene encoding APP is located on chromosome 21q21.3. It is a highly conserved gene containing 18 exons and spanning 290 kilobases [16]. APP protein is a ubiquitously expressed single-pass type I transmembrane protein, with a large extra-membranous N-terminal region, a single transmembrane domain and a small cytoplasmic C-terminal tail [17, 18]. APP proteins range from 365 to 770 amino acids due to different splicing isoforms [9, 19].

Alternative splicing of transcripts from the single *APP* gene results in several isoforms of the gene product. APP695, APP751 or APP770 are the most common APP isoforms [20, 21]. APP695 is preferentially expressed in the central nervous system. APP751 and APP770 expressed both in the peripheral and central nervous systems. The ratios of APP770 mRNA and APP770-plus-APP751 mRNAs were increased significantly in AD brain [21]. Both APP751 and APP770 contain the Kunitz protease inhibitor (KPI) domain, and APP770 also contains an OX-2 domain [22]. APP695 on the other hand, lacks both of these domains. The up-regulation of the KPI-containing APP isoforms has been reported in the brains of AD patients and could be associated with the disease’s progression [23]. Except for the protease-inhibitor role of the KPI domain, no other definite functional differences have been found in the different APP isoforms [20, 24, 25]. Other isoforms are referred to as leukocyte-derived APP (L-APP). L-APP mRNA is either seldom or never expressed in the central nervous system tissues [26].

As we known, extracellular deposition of the Aβ, the major constituent of senile plaques, is derived from the APP by proteolysis. There are at least two major pathways (the non-amyloidogenic and amyloidogenic pathways) leading to proteolytic cleavage of APP by α-, β-and γ-secretases [27]. The non-amyloidogenic pathway, a proteolysis process including α- and γ-secretases, results in nonpathogenic soluble α-Cleaved soluble APP (sAPPα) and a membrane-bound α-C-terminal fragment (αCTF). In this pathway, the APP protein is cleaved by α-secretase in the middle of the Aβ sequence, resulting in the release of sAPPα. Subsequently, the remaining αCTF is cleaved by γ-secretase, resulting in the release of the P3 peptide and the amyloid intracellular domain (AICD) [27–29]. In the amyloidogenic pathway, which is common in neurons, the APP protein is cleaved by β-secretase at the 1st residue or at the 11th residue of Aβ sequence, resulting in the release of soluble β-Cleaved soluble APP (sAPPβ). Next, the remaining βCTF in the membrane is cleaved by γ-secretase, leading to a mixture of Aβ peptides of different lengths (38 to 42 amino acids). Aβ1-40 (around 90%) and Aβ1-42 (around 10%) are the two major Aβ peptides [30, 31].

#### The function of APP in the central nervous system

APP is essential for physiological processes such as neural proliferation, migration, differentiation, plasticity and synaptogenesis [32–36]. Acutely knock down *APP* in the developing cortex of Sprague Dawley rats revealed that neuronal precursor cells in the embryonic cortex require APP to migrate correctly into the nascent cortical plate [32]. APP was also shown to play an important role in the cell cycle progression of neural stem cells through its interaction with APP binding protein-1 [33]. APP regulates netrin-1-mediated commissural axon outgrowth [34]. The functions of reelin and integrins on neurite development, neuronal migration and synapse functions may also be modulated by APP [35].

Genetically modified mice have shown that mice lacking *APP* are viable, fertile and exhibit a relatively mild phenotype, including a reduced body and brain weight, as well as several neurological symptoms such as reduced grip strength, deficits in spatial memory and increased susceptibility to seizures [37–40]. These symptoms are probably related to changes at the cellular and network level, including decreased dendritic spines, reduced hippocampal long-term potentiation and altered short-term neural plasticity [37, 41–43]. The development of a neuronal circuit requires the maintenance of synaptic homeostasis demands on a coordinated proteomic response at both pre- and postsynaptic sites. A very recent study showed that APP proteins are one of the unique sets of proteins regulating proper presynaptic physiology [44, 45]. Synaptic homeostasis entails a stable but plastic network and neuronal circuit. The combination of bioinformatics tools and biochemical approaches has shown that APP is a structural and functional regulator in a context-sensitive manner within the hippocampal active zone network [46].

During neuronal differentiation, *APP* expression remained stable, whereas the proteolytic processing of APP changed at various stages of differentiation [47]. sAPPα was secreted in the early stages of differentiation (neuronal progenitors), and sAPPβ was usually secreted after the production of deep-layer neurons. A recent study has shown that sAPPα, a neurotrophic fragment released from the metabolites of APP, is also important to synaptogenesis [36, 48].

#### The mutation spectrum of APP

To date, about 50 pathogenic mutations of *APP* have been reported (Alzheimer Disease and Frontotemporal Dementia Mutation Database. http://www.molgen.ua.ac.be/admutations.), most of which affect the proteolysis of APP in such a way that Aβ1-42 levels are changed relative to other Aβ isoforms [49–54]. Most pathogenic mutations of *APP* occur near the β-secretase cleavage site (amino acids 670aa-682aa), near the γ-secretase cleavage site (amino acids 713aa-724aa) or in the Aβ sequence (amino acids 692aa-705aa) of the APP protein [55]. The mutations (D7H, E682K and K16N) that affect the β-secretase cleavage site cause a significant increase in the total levels of Aβ and Aβ1-42/40 [56–58]. The mutations (T714I, V715M, V715A, I716V, V717I, V717L, L723P and K724N) that affect the γ-secretase cleavage site cause an increased relative ratio of Aβ42 to Aβ40 [59–61]. The mutations clustered around the central hydrophobic Aβ core near the α-secretase cleavage site (E693G Arctic mutation, E693K Italian mutation, D694N Iowa mutation and 692G Flemish mutation) could result in a variety of polymorphic aggregates in a mutation-dependent manner, and could disrupt the integrity of the bilayer [62, 63].

The missense mutation V717I, the first described and best characterized of all *APP* mutations, is located in the transmembrane domain near the γ-secretase cleavage site, and affects both the β- and γ-secretase cleavage of APP protein [64]. This mutation changes the initial cleavage site of γ-secretase, which results in the increase of both Aβ42 and Aβ38 peptides. The initial clinical phenotype for a Chinese AD patient carrying the V717I mutation is characterized by prominent early affective symptoms, executive dysfunction and disorientation in comparison with Western patients [65]. This finding shows that the phenotypes of AD patients with identical *APP* mutations are affected by ethnic differences, environment or other unknown factors [65, 66].

Other than these dominant mutations, a recessive amino acid deletion mutation (E693Delta), which is more resistant to proteolytic degradation, and a recessive missense mutation (A673V) with a dominant negative effect on amyloidogenesis haves been reported [50, 67]. The E693Delta mutation has been suggested as a cause of dementia because of enhanced formation of synaptotoxic Aβ oligomers [67]. The A673V mutation shows highly amyloidogenic effect in the homozygous state and anti-amyloidogenic effect in the heterozygous state [50]. In the same amino acid position, another mutation (A673T) enriched in the Icelandic population is reported to be a protective variant [68]. This variant is adjacent to the β-secretase cleavage site of APP, and results in a reduction of approximately 40% in the formation of amyloidogenic peptides *in vitro*. This variant also protects against cognitive decline in elderly people without AD.

In addition, genomic duplications of variable size containing *APP* have also been identified in autosomal dominant EOAD [69]. In contrast to missense mutations, which show a near-complete disease penetrance, genomic duplications of *APP* are rare and display a higher variability in the age of onset. Moreover, the dementia of patients with *APP* duplication show a virtually complete penetrance by the age of 65 [69, 70]. Their phenotype is not associated with the size of the duplication. Patients with a duplicated *APP* gene are more often affected by seizures compared with patients suffering from other missense variants [71].

### Presenilin 1 (PSEN1)

#### The gene and protein structure of PSEN1

After the discovery of the *APP* mutations that cause AD, *PSEN1* was identified as the most common cause of autosomal dominant EOAD. *PSEN1* mutations account for 70% to 80% of autosomal dominant EOAD cases [54, 72]. *PSEN1* is located on chromosome 14q24.2. It consists of 12 exons encoding a 467-amino-acid protein, which has 9 C-terminal transmembrane domains locating to the lumen/extracellular space [73]. Full-length PSEN1 is an inactive precursor and could be transformed into a heterodimer composed of a 30 kDa N-terminal fragment (NTF) and a 20 kDa C-terminal fragment (CTF) by an endoproteolytic cleavage within hydrophobic domain 7 in a large cytosolic loop [74]. The NTF/CTF heterodimers are the active forms of PSEN1 as well as the predominant forms detected in cells, whereas the full-length proteins not targeted for the cleavage pathway are rapidly degraded. PSEN1 endoproteolysis may be an important step in the process of activating the γ-secretase complex. Furthermore, the NTF/CTF heterodimers may form the catalytic core of the γ-secretase complex [75, 76]. The two catalytic aspartate residues (Asp257 at NTF and Asp385 at CTF) in PSEN1 are important for the activity of γ-secretase, and each mutation of the two conserved aspartates could abolish the γ-secretase activity [75, 77, 78].

### The function of PSEN1 in the central nervous system

PSEN1 is one of the four core proteins (others include nicastrin, anterior pharynx-defective 1 and presenilin enhancer 2) in the γ-secretase complex, which is considered to play an important role in the generation of Aβ from APP [79]. Some studies have shown that γ-secretase activity in hippocampal neurons lacking PSEN1 is inhibited obviously than wild-type neurons [80]. The *PSEN1* knockin studies showed that PSEN1 plays an important role in synaptic plasticity [81–83]. The knockout mice exhibit perinatal lethality with developmental defects and impaired neurogenesis. Conditional *PSEN1* knockout studies have shown that the loss of PSEN1 activity causes synaptic dysfunction, memory impairment and age-dependent neurodegeneration in the excitatory neurons of the postnatal forebrains of mice and a pronounced deficiency in enrichment-induced neurogenesis in the dentate gyrus [84–86]. These studies have suggested that PSEN1 may play an important role in promoting and maintaining memory and neuronal survival [87, 88]. At the cellular level of the knockout model, this inactivity has no effect on evoked neurotransmitter release, short-term plasticity or the apparent calcium dependence of the evoked release [89]. This physiological function maybe associated with the interaction with the pre-synaptic protein synaptotagmin 1 (Syt1), a calcium sensor in synaptic vesicle exocytosis [90]. The binding between PSEN1 and Syt1 could regulate synaptic vesicle trafficking along neuronal processes, promoting exocytosis and neurotransmitter release and leading to neurodegeneration in the affected circuits in neurodegenerative diseases [90].

### The mutation spectrum of *PSEN1*

Patients with *PSEN1* mutations typically have an earlier age of onset, with symptoms starting an average of 8.4 years earlier than in *APP* mutation carriers (an average of 42.9 years of age vs. 51.3 years) and 14.2 years earlier than in *PSEN2* mutation carriers (an average of 57.1 years of age) [54]. Other studies have shown that very early-onset AD (VEOAD), which starts before the age of 35 years, is almost entirely caused by *PSEN1* mutations [91, 92]. Like *APP*, mutations in the promoter region of *PSEN1* also were found to be associated with increased risks of EOAD, perhaps due to an alteration of *PSEN1* gene expression with a subsequent influence on Aβ load [93, 94]. Seizures and myoclonus is common feature of autosomal dominant EOAD, which was associated with *PSEN1* mutation [95, 96].

To date, more than 200 pathogenetic mutations have been described in *PSEN1* throughout the world, of which 70% mutations occur in exons 5, 6, 7 and 8 (Alzheimer Disease and Frontotemporal Dementia Mutation Database. http://www.molgen.ua.ac.be/admutations.). The majority of *PSEN1* mutations in EOAD are missense mutations, which cause amino acid substitutions. Mutations in the transmembrane domains 2 and 4 are associated with an earlier age of onset and death than those in the transmembrane domains 6 and 8 [97]. *PSEN1* mutations affect Aβ production through a relative increase in the ratio of Aβ42/Aβ40 [98, 99]. Aβ42 is more prone to forming amyloidogenic aggregates in brain than Aβ40 [100]. Investigations into the effects of *PSEN1* mutations on γ-secretase have shown that around 90% of those reported mutations lead to the reduced production of Aβ42 and Aβ40, while the majority of mutations lead to increased Aβ42/Aβ40 ratios *in vitro*. There is no statistically significant correlation between the Aβ42/Aβ40 ratio affected by *PSEN1* mutations and the mean onset age of patients carrying the mutation [98]. These patients with *PSEN1* mutations usually have higher amounts of total Aβ deposits in the brain than sporadic AD patients. Research into *PSEN1* mutation mechanisms that affect APP processing by γ-secretase has had conflicting results in different experimental systems, which may be affected by endogenous PSEN1, PSEN2 or other components of γ-secretas [101, 102].

### Presenilin-2 (PSEN2)

#### The gene and protein structure of PSEN2

Less than a year after mapping *PSEN1*, another gene encoding the transmembrane protein PSEN2 showed a significant association with AD. *PSEN2*, located on the long arm of chromosome 1 (1q42.13), has a nearly 60% homology to *PSEN1* [103, 104]. It consists of 12 exons encoding a 448-amino-acid protein that is predicted to consist of 9 transmembrane domains and a large cytoplasmic loop domain between the 6th and 7th domains [104, 105]. *PSEN2* has two isoforms. Isoform 1 is found in the placenta, skeletal muscle and heart, while isoform 2, which lacks amino acids 263–296, is found in the brain, heart, placenta, liver, skeletal muscle, and kidney [103, 104, 106–108].

PSEN2 is also one of the four core proteins in the γ-secretase complex and provides the catalytic activity of the complex. *PSEN2* has been poorly studied and is considered to be a compensatory partner of *PSEN1* [109]. In contrast to the broadly distributed PSEN1, PSEN2 is known to be mainly restricted to a specific subcellular compartment, such as late endosomes and lysosomes [110]. The more restricted localization of the PSEN2 contributes to the intracellular pool of Aβ peptide previously associated with an early event in AD [111].

#### The function of PSEN2 in the central nervous system

Some controversial results have suggested that PSEN2 has a role in apoptosis [112–115]. Research into PSEN2-overexpressing Neuro2a cells has shown that PSEN2 could result in reduced viability and condensed chromatin. It could also affect the expression of Bax associated with apoptosis, but not the expression of p53 and PSEN1 [116]. Lots of studies suggest that PSEN2 proteins in the immune system have a variety of biologically important roles. The loss of presenilin function is associated with neuroinflammation and neurodegeneration [84, 85, 117]. PSEN2 could increase Aβ-induced classical proinflammatory cytokines such as IL-1β, IL-1α and TNF-α in knockout microglial cells [118]. PSEN2 could play a potential role in modulating lipopolysaccharide-mediated immune responses [119]. PSEN2 also modulates endoplasmic reticulum-mitochondria coupling in the presence of mitofusin-2, which is crucial for the regulation of various physiological and pathophysiological processes [120].

#### The mutation spectrum of PSEN2

Unlike the *PSEN1*, mutations in the *PSEN2* gene are extremely rare. Less than 40 mutations in *PSEN2* have been identified (Alzheimer Disease and Frontotemporal Dementia Mutation Database. http://www.molgen.ua.ac.be/admutations.). *PSEN2* mutation might increase γ-secretase activity. The known pathogenic mutations of *PSEN2* lead to a significant decrease in extracellular Aβ40, an increase Aβ42 and a dramatic rise in the Aβ42/40 ratios. This change is more pronounced in the intracellular pool of Aβ. Except for two frame shift mutations, Glu126fs and Lys306fs, the others are nonsynonymous substitutions [54]. Familial AD with *PSEN2* mutations have a later age of onset, longer disease duration compared with families with *PSEN1* mutations [121]. Except in the case of familial AD, some *PSEN2* mutations are associated with other disorders, such as dementia with Lewy bodies, frontotemporal dementia, breast cancer, dilated cardiomyopathy and Parkinson’s disease with dementia (PDD) [105]. The penetrance of the disease in AD patients with *PSEN2* mutations is variable and the age of onset ranges widely, from 40 to 80 years of age [105, 122, 123]. Only 17 of the 38 are predicted to be disease-causing mutations. Ten of the mutations are not pathogenic and the others are still unclear. The mutations T122P, N141I, M239I and M239V cause the increase of Aβ amount [124]. The mutations T122R, S130L and M239I were found to alter calcium signaling [125, 126].

### Genetically unexplained EOAD

Research into the genetics of EOAD has made great progress, but pathogenic mutations in *APP*, *PSEN1* and *PSEN2* can only explain a small fraction of EOAD families. The large number of genetically unexplained EOAD patients suggests that the additional disease-causing genes have not yet been identified. In the last few years the next generation sequencing technologies, such as whole genome sequencing and whole exome sequencing, offer new insights into the genetic etiology of EOAD.

The homozygosity for the *APOE* ε4 allele, as a major genetic risk factor for LOAD, was shown to be an independent genetic factor that significantly increases the risk of EOAD [127]. But, unlike mutations in *APP*, *PSEN1* and *PSEN2*, the *APOE* ε4 allele was not considered a significant cause of EOAD and was only a risk factor [128]. Some research into EOAD families using next-generation sequencing technology has identified some genes that could be candidates for causing EOAD, such as *TYROBP*, *NOTCH3* and *SORL1* [129–132]. The use of exome sequencing in EOAD patients identified some *TYROBP* variants might contribute to the risk of EOAD [129]. TYROBP might be involved in Aβ turnover. The partial loss of the TYROBP signaling pathway has been suggested to be responsible for the neurological phenotypes of cognitive decline. The whole exome sequencing technology identified a mutation in *NOTCH3* is associated with AD [130]. *NOTCH3* has been associated with cerebral autosomal dominant arteriopathy with subcortical infarcts and leukoencephalopathy (CADASIL), a dementia disorder which clinical phenotype overlaps with EOAD. Dysfunctional Notch signaling may also induce or inhibit genes that are important in the pathogenesis of AD or in PSEN function. Several mutations in *SORL1*, a sorting protein-related receptor gene involved in the trafficking of APP and Aβ, was found in autosomal dominant EOAD patients without mutation on the known genes (*APP*, *PSEN1* and *PSEN2*) by exome sequencing and whole genome sequencing studies [131, 132]. The screening of *NOTCH3*, *SORL1* and *TYROBP* in larger patient/control groups would help to define the contribution of rare genetic variants in the three candidate genes to the etiology of EOAD.

### Summary

AD is one of the most challenging disorders and is characterized by dementia that typically begins with the progressive recession of memory and slowly becomes more severe and incapacitating. Understanding the genetics of AD may lead to its early detection, prevention and treatment. Compared with LOAD, learning more about the genetics of the rare, early-onset familial form of AD could result in a better understanding of the pathophysiology of the disease. In this review, we have summarized the main genetic aspects of EOAD and their role in the physiological function of the nervous system and the pathological function of disease mechanisms in EOAD patients. We have identified 3 high-penetrant EOAD genes (*APP*, *PSEN1* and *PSEN2*) by genetic linkage studies and the gene cloning method in exceptionally large and informative monogenic pedigrees. The *APOE* ε4 allele also increased the risk of EOAD in carriers of at least one ε4 allele, and was a significant risk factor for EOAD independent of other genetic factors. Thanks to new high-throughput sequencing technologies, the systematic screening of the causal genes for dementia in both familial and sporadic patients has uncovered some other candidate genes. Investigations into the missing genetic etiology in unexplained EOAD patients still has a vast potential to discover new and crucial genetics aspects that will lead to a better understanding of the pathological mechanisms leading to AD. Moreover, these findings will accelerate the development of new therapeutic strategies for preventing, stopping and even reversing AD.
